# Predictors of Land-Based Activity Participation in a National Representative Sample of Indigenous Individuals Living Off-Reserve

**DOI:** 10.3390/ijerph19138029

**Published:** 2022-06-30

**Authors:** Elaine Toombs, Jessie Lund, Aislin R. Mushquash, Christopher J. Mushquash

**Affiliations:** 1 Department of Psychology, Lakehead University, 955 Oliver Road, Thunder Bay, ON P7B 5E1, Canada; jlund@lakeheadu.ca (J.L.); aislin.mushquash@lakeheadu.ca (A.R.M.); chris.mushquash@lakeheadu.ca (C.J.M.); 2 Dilico Anishinabek Family Care, Fort William First Nation, Thunder Bay, ON P7B 5E1, Canada; 3 Department of Psychology, Northern Ontario School of Medicine University, Thunder Bay, ON P7B 5E1, Canada; 4 Thunder Bay Regional Health Sciences Centre, Thunder Bay, ON P7B 5E1, Canada; 5 Thunder Bay Regional Health Research Institute, Thunder Bay, ON P7B 5E1, Canada

**Keywords:** First Nations health, Indigenous mental health, land-based treatment, cultural treatment, population-level health, public health

## Abstract

This study examined data from the 2017 Aboriginal Peoples Survey to consider predictors of land-based activity engagement. We hypothesized that higher self-reported mental and physical health scores, an increased sense of cultural belonging, living in a rural community, and no prior individual or family history of residential school attendance would predict a higher frequency of land-based activity engagement among First Nations individuals living off-reserve. Results from linear regression analyses suggested that an increased sense of cultural belonging, being male, and living in a rural community with a population of less than 1000 people were significant predictors of the frequency of land-based activity engagement. With these preliminary findings, further research can explore how physical and mental health outcomes influence the frequency of land-based activity engagement, in addition to how community-specific indicators may promote higher frequency of these activities, particularly among First Nations individuals living off-reserve.

## 1. Introduction

Many Indigenous cultures have long shared teachings emphasizing the importance of place and engaging with nature to promote mental, spiritual, emotional, and physical wellbeing. These teachings, although specific to community, region, and cultural identity, have been communicated across generations, and prioritize the deep spiritual connectedness to land experienced by many Indigenous people. The overall benefit is understood within Indigenous teachings, and is considered to be integral to individual and community health and wellbeing.

Land-based approaches to wellbeing and healing are activities that emphasize being on and connecting with the land and nature in a culturally or spiritually significant way. For Indigenous people, one’s sense of self can be informed by one’s sense of place [1,2,3,4]. This can be one’s interconnection with spirit, land, people, animals, and plants, including how such entities co-exist together in a shared environment. Land-based activities emphasize the connectivity within these relationships, and situate an individual within meaningful culturally and spiritually significant contexts [5]. It is this spiritual significance of engaging in land-based activities that differentiates these approaches from other nature-focused activities or programs, which can have similar psychological benefits, but do not emphasize connection to place or cultural significance [6]. For example, engaging in these approaches within ancestral land that has been used for the same traditions across generations can bolster the purpose and meaning of these culturally specific activities [7].

Given that Indigenous conceptualizations of wellness can be rooted to a sense of place, promoting culture through land-based activities is particularly relevant to present-day efforts to reconcile Indigenous disparities in health. Currently, in Canada, Indigenous people experience poorer mental and physical health outcomes when compared to non-Indigenous populations. For example, Indigenous adults in Canada have a lower life expectancy than non-Indigenous adults [8], with Inuit men living approximately 15 fewer years than non-Indigenous men in Canada [9]. Although rates differ across regions, Indigenous populations have higher rates of suicide [10], are more likely to be hospitalized for acute substance-use-related concerns [11], and are more likely to experience adversity in childhood, such as exposure to intimate partner violence [12], when compared to non-Indigenous people. Revitalizing land-based activities has been championed as one mechanism to rectify the detrimental effects of longstanding historical and present-day discrimination experienced by Indigenous people that contributes to Indigenous health disparities [13]. Land-based activities serve as a practice that can reclaim Indigenous identity in response to a longstanding history of colonial and racist institutional policies and procedures that have aimed to assimilate Indigenous people by eliminating access to language, culture, and traditions.

### 1.1. Recent Land-Based Activity Research

Recent research has explored how land-based activities can promote wellbeing, resilience, and health outcomes among Indigenous adults [14,15,16]. *Culture-as-treatment* models are viewed as integral to Indigenous-specific interventions for mental health and wellbeing, and one way to address current disparities between Indigenous and non-Indigenous mental and physical health indicators [17,18,19,20]. These interventions prioritize Indigenous-specific approaches to health that authentically address the holistic relationships of mental, emotion, physical, and spiritual wellbeing, encapsulated by the four directions of the medicine wheel as mind, emotion, body, and spirit. Land-based activities such as cultural camps [15,21,22,23], canoeing [24,25], harvesting practices (including hunting, trapping, and gathering plants or medicines) [26,27,28], and other land-based ceremonies promote improved mental health outcomes among Indigenous people.

To date, the majority of studies examining land-based activities for mental health promotion within Indigenous communities use either qualitative or mixed methods. Evaluation of these community-led programs has been challenging, as developed interventions are specific to each community context and, as such, have different logistical needs, and offer different approaches to bolster connection with cultural knowledge [29,30]. The outcomes of each program also vary, with many land-based programs in the literature appearing to be for youth or young adults, or focused on those with substance-use disorders. Quantitative studies documenting outcomes of land-based or cultural activities remain limited, and at times their results are conflicting. Among a sample of Indigenous youth, valuing cultural activities was associated with decreased odds of both alcohol consumption and episodes of heavy drinking [31]; however, another study of Indigenous youth reported that increased engagement in hunting, fishing, and/or trapping was a significant predictor of heavy alcohol use among Indigenous individuals [32]. It is suggested that, in the latter study, sex-based differences in participants’ alcohol use and land-based activity participation may have confounded the results, such that males in the study were more likely to engage in hunting, fishing, or trapping, but were also more likely to be heavy drinkers.

### 1.2. Cultural Approaches to Indigenous Wellbeing

Many Indigenous models of wellness promote a holistic understanding of wellbeing that emphasizes a balance of the four directions, modeled through the four quadrants of the medicine wheel that represent mental, physical, emotional, and spiritual wellbeing [16]. The First Nations Mental Wellness Continuum Framework (FNMWCF) [33] is a model of wellness conceptualized by First Nations communities within Canada, and describes four facets of mental wellness, corresponding to these four quadrants of the medicine wheel. This model suggests that increased hope, belonging, meaning, and purpose correspond to increased mental wellness within an individual. These aspects are bolstered by intersecting wellness of family, community, society and, finally, culture, which is proposed to encompass every other component within the model. This model suggests that culture is embedded in every component of wellness and, thus, must be integrated at all levels of care across institutions and practices to promote Indigenous wellbeing [33].

Bolstering various aspects of access to—and an individual connectedness to—culture has been associated with increased resilience within Indigenous communities [20,34]. The benefit of culture as both a process one actively engages in and/or an outcome indicator has been conceptualized in a variety of ways within Indigenous health research. These include various processes and outcomes that relate to engagement in culture, including promoting cultural identity [35,36,37], cultural connectedness [38,39,40,41], cultural efficacy [42], and cultural continuity [43,44,45]. For example, among residential school survivors, connectedness to culture has been associated with better mental health outcomes [39]. Within rural areas in Australia, Indigenous language use has been associated with lower rates of suicide, particularly among individuals experiencing increased racial discrimination [38]. Valuing cultural identity has been associated with less alcohol use among First Nations youth [31]. Promoting various cultural practices, including Indigenous language use, has been associated with similar outcomes [46]. Although these components represent distinct protective factors, all are promoted through engagement in some types of cultural practices.

With respect to promoting an individual sense of belonging, the FNMWCF aligns belonging with both community and culture [33]. Given the multifaceted nature of community-based approaches that promote cultural practice, it is likely that engagement in these activities has compounded and intertwined benefits across many aspects of wellness. As such, it is difficult if not impossible to parse out the specific mechanisms of action within such practices. For example, engagement in land-based activities has been identified as an approach that promotes Indigenous health and wellbeing; however, such practices can also be facilitated by the development of secondary protective factors that can foster resilience among Indigenous populations. For example, increasing cultural connectedness has been associated with bolstering resilience in Indigenous youth [41]. As having a strong cultural identity may bolster regular engagement in land-based practices, these components can foster wellbeing in a twofold manner: (1) from the sole benefit of engaging in land-based practices that promote wellbeing, and also (2) various strengths prior to frequent engagement in these practices, such as a strong cultural identity, cultural connectedness, community engagement, or cultural belonging, among others. More research is required to determine how specific aspects of these practices—including the frequency of engagement in cultural activities—relate to mental health and physical health, among other aspects of cultural belonging within Indigenous communities.

### 1.3. Study Purpose

The purpose of this study was to explore predictors of frequency of engagement in land-based activities among First Nations individuals living off-reserve, using the Aboriginal Peoples Survey (APS) 2017 [47]. Although previous research has examined some predictors of engagement in hunting, fishing, or trapping, related to age, sex, household composition, and physical health [10], predictors of the frequency of land-based activities with respect to mental health and self-reported cultural belonging have yet to be explored. Land-based activities are often used within *culture-as-treatment* models to promote positive mental health outcomes among Indigenous individuals [20]; however, research to date has not examined the inverse of this relationship, i.e., whether those individuals with higher self-reported mental and physical health are more likely to report a higher amount of engagement in traditional activities. Previous predictors of any type of engagement in land-based activities have typically been associated with increased mental wellbeing [20]. It was expected within the current study that those who engaged in activities more frequently would report higher mental and physical health ratings, when age, sex, and rural location were entered as covariates. Given previous literature describing any participation in land-based activities [10], we hypothesized that residing in a rural community, being older, and being male would predict the frequency of land-based engagement when initially entered in our linear regression model.

**Figure 1:**
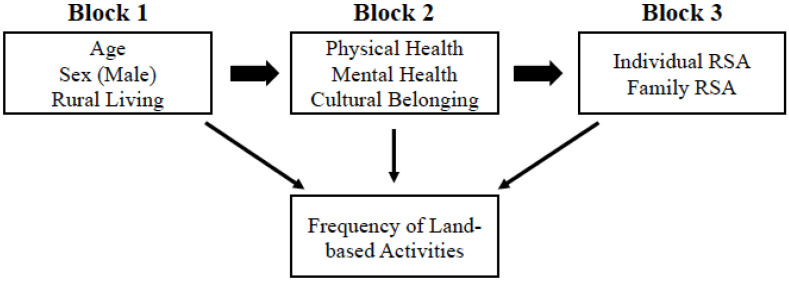
Predictors of engagement in land-based activities.

We also explored whether increased cultural belonging and/or having no prior history of individual residential school attendance would predict a higher likelihood of engaging more frequently in land-based activities. Given the historical and present-day disruption to Indigenous access to and participation in land-based activities, we hypothesized that reporting a higher sense of cultural belonging and having no prior history of family or individual residential school attendance would be associated with increased frequency of engaging in these cultural practices (see Figure 1 for a description of our proposed model). Although the benefits of engaging in traditional activities are well documented [20], determining who engages in such activities more frequently can help shape a preliminary understanding of specific components that contribute to Indigenous wellbeing. By understanding who frequently engages in land-based activities, we can also better contextualize potential barriers to these activities for future land-based treatment or wellness models.

## 2. Methods

### 2.1. Use of Population-Level Data to Explore Engagement in Land-Based Activities

The APS 2017 provides an opportunity to explore the relationships between engagement in land-based activities and health outcomes among various communities of Indigenous people in Canada [47]. Currently on its fifth iteration, the APS is a cross-sectional study of Indigenous health outcomes, sociodemographic characteristics, and cultural engagement, among other variables of interest, for First Nations individuals living off-reserve, as well as Métis and Inuit individuals living across Canada. Descriptive analyses across four iterations of the APS (2001, 2006, 2012, 2017) indicate that by 2017, the prevalence of hunting, fishing, or trapping activities among Indigenous individuals living off-reserve had decreased by approximately 10% [10]. Most recently, in 2017, approximately 47% of First Nations individuals living off-reserve engaged in hunting, fishing, trapping, or gathering wild plants or berries in the year prior. Rates of engagement in hunting, trapping, or fishing (excluding plant gathering) were lower in 2017 than in previous years (33% of individuals), declining from 37% in 2006; however, rates of plant gathering had stayed relatively stable. The most common reasons endorsed by survey respondents in 2012 for engaging in these activities were for personal or family use, or as a pleasure or leisure activity, with noted barriers related to time, costs of supplies or equipment, lack of people to engage in the activity with, and location [10].

### 2.2. Participants

Participants were selected from survey respondents of the APS (2017) aged 15 years or older. Individuals who responded “First Nations” to the survey item “Are you First Nations, Métis, or Inuk?” were included in these analyses. Relevant participant demographic information is described in Table 1.

### 2.3. Variables from the APS (2017) Dataset

#### 2.3.1. Demographics

Age and sex variables were obtained from the APS 2017. A single-item measure of rural living was calculated by transposing those who indicated that they lived in a rural community with a population under 1000 people, and creating a dummy variable coded as “1” or “0” based on rural living status.

#### 2.3.2. Frequency of Land-Based Activities

Two variables from the APS—“In the past twelve months, did you hunt, fish, or trap?” and “in the past twelve months, did you gather wild plants, for example, berries, rice or sweet grass?”—were used to compose a measure of land-based activity frequency. For those who answered “no” to either of these items, responses were entered as “Never” and coded as a 0, and combined with a second set of survey items that were asked to every participant who answered “yes” to these questions. For those who endorsed either (1) frequency of hunting, fishing, or trapping, or (2) gathering wild plants, they were answered to clarify the frequency of each activity on a 5-point Likert-type scale. These survey items were initially answered as 1 = every day, 2 = a few times a week, 3 = once a week, 4 = at least once a month, or 5 = less than once a month. In initial exploratory analyses using Pearson’s two-tailed correlations, these variables were highly correlated (*r* = 0.83). These responses were then reverse-coded, and the highest score of the hunting/fishing/trapping and the plant gathering variables was used to represent one’s overall frequency of land-based activities, and transposed with the initial scores of those individuals who indicated that they never engaged in land-based activities. This final variable therefore consisted of six-item Likert-type responses ranging from 0 = “never” to 5 = “every day”.

#### 2.3.3. Self-Reported Health

Two variables—self-reported physical and mental health—were used directly from the APS. Participants rated both physical and mental health on a 5-point Likert-type scale, ranging from poor to excellent.

#### 2.3.4. Cultural Belonging

Cultural belonging was operationalized using one item entitled “I have a deep sense of belonging to my First Nations/Aboriginal group”, and was rated on a five-point Likert-type scale ranging from strongly disagree to strongly agree. Items were reverse-coded to reflect higher scores being associated with higher scores of cultural belonging.

#### 2.3.5. Residential School Attendance

Individual residential school attendance (RSA) was a single-item variable. Family residential school attendance was generated from two variables that asked about parent and grandparent RSA. To assess whether multiple generations of RSA influence engagement in land-based activities, a composite variable of family RSA was created, with 0 indicating no family history of RSA, 1 indicating parent RSA history, and 2 indicating parent and grandparent RSA.

### 2.4. Analytic Procedure

Access to the 2017 APS analytic file was provided by the Canadian Research Data Centre Network following an approved request and documented exemption from the Lakehead University Research Ethics Board for use of secondary data, consistent with the Tri-Council Policy Statement: Ethical Conduct for Research Involving Humans [48]. To align with Statistics Canada regulations for use of the 2017 APS analytic file, all data were weighted by person to represent not only themselves as an individual, but also others within the population who were not sampled. Each individual case (i.e., person) had a calculated weight that was used to calculate the number of people each individual case represented, based on intersecting population-level demographic characteristics such as age, sex, Indigenous status, and region [47]. Although the initial analyses were completed with *n* = 10,030 respondents, weighted analysis corresponded with survey data to represent a total population of *N* = 491,010. The reliability of these weighted analyses was assessed by generating confidence intervals from 1000 bootstrapped weights provided in the APS analytic file. Finally, in accordance with Statistics Canada policies, any cell counts less than or equal to 10 individuals were removed to correspond with respondent confidentiality protocols, and frequency counts were rounded to the nearest 10.

## 3. Results

Predictors of land-based activity frequency were entered into a linear regression model first by age, sex, and rural location, followed by mental and physical health variables (i.e., self-reported mental health, physical health, and cultural belonging) and, finally, by RSA (individual and family). See Table 2 for these linear regression results. Among predictors of land-based activity for First Nations individuals living off-reserve, cultural belonging, living in a rural community, and being male were significantly associated with increased frequency of engagement in land-based activities. Contrary to our hypotheses, self-reported ratings of both physical and mental health were not associated with increased engagement in land-based activities, nor did a prior history of individual or family RSA predict less engagement.

## 4. Discussion

The purpose of the present study was to explore predictors of land-based activity engagement among First Nations individuals living off-reserve. An increased sense of cultural belonging was a strong predictor of the frequency of engagement in land-based activities, which is congruent with previous models of Indigenous mental wellness such as the FNMWCF [33]. The results in the present study support the relationships described in the FNMWCF model—specifically, that belonging fostered through culture is a significant predictor of engagement in land-based activities. This finding supports existing models that describe how increased engagement in cultural practices and identities among Indigenous communities is protective against adverse physical and mental health outcomes, including racial discrimination, colonialism, and historical trauma [33,38,39,49]. For example, cultural resilience has been significantly associated with mental, physical, emotional, and spiritual wellbeing, including self-reported physical health [49].

The results of this study also showed that those living in remote or rural communities (i.e., with a population of under 1000 individuals) were more likely to engage in land-based activities and report better cultural connectedness. These data correspond with studies of First Nations individuals living on-reserve as well, showing that residing in such a community (often less than 1000 individuals) also predicts engagement in land-based activities. Geographical location—particularly rural and remoteness (associated with limited access to resources, health services, and other challenges)—can complicate understandings of engagement in health behaviors in population-level data. It remains difficult to quantitatively interpret relationships between predictors of engagement in land-based activities and subsequent health outcomes in a way that is authentic to Indigenous ways of knowing and community-based values in population-level datasets. For example, previous analysis of community wellbeing data and language use demonstrated that increased language use was associated with decreased Community Wellbeing Index (CWI) scores, comprising education, income, housing, and engagement in the labor force; however, these results were likely confounded by the geographical location of communities [50]. Furthermore, land-based knowledge and engagement may vary significantly by community due to the varying extent to which the reserve system interrupts access to land that was historically accessed. With respect to predicting engagement in land-based activities, careful understanding of community-based values, culture, and traditions, as well as historical geographical patterns of land-based activity engagement, are required to generate useful predictive models of who engages in land-based activities, which can inform broader understandings of community wellness and mental health promotion.

A similar rationale may be extended to the present study, such that rural and remote communities in Canada, including First Nations reserves and other communities, tend to experience unique challenges to mental and physical healthcare delivery. Those residing in remote or rural regions tend to have decreased access to facets that promote social determinants of health [51]. For example, rural-residing individuals are more likely to report health disparities [52] that have been attributed to decreased socioeconomic status [53], having decreased access to primary healthcare providers or specialists [54,55], and reduced access to educational or employment opportunities [56]. In the present study, decreased health outcomes can intersect with experiences of these social determinants, which can affect the way in which First Nations individuals have access to and engage in land-based activities.

Neither individual nor family RSA was significantly associated with decreased frequency of cultural engagement. Although it is possible that recent efforts of reconciliation and reparative cultural engagement have mitigated the influence of these relationships, it is also plausible that ongoing experiences of colonialism—including ongoing land and water pollution [57,58], reserve systems and denial of Indigenous land rights or sovereignty [59], and inaccessibility of traditional knowledge—may continue to affect the frequency of land-based activities or such practices’ influence in promoting health [60], regardless of history of RSA. For example, disruptions to intergenerational traditional knowledge sharing of culturally relevant food storage and preparation practices can increase exposure to biological contamination of food and depletion of resources from non-traditional hunting or fishing practices, which would ultimately reduce engagement in such activities [61].

Higher self-rated physical and mental health were not associated with increased engagement in land-based activities when entered into our regression model. Our study’s results did not support a linear relationship between health and land-based activity engagement analyses. It is possible that there may be a bidirectional relationship between individuals who use land-based activities to promote mental and physical health, not explored in the present study, as these strategies are used for both healing (i.e., those with lower mental health ratings) and general wellness promotion (i.e., likely those with higher mental health ratings). For example, a previous analysis of the use of traditional healing practices found that engagement in these practices was associated with poorer self-reported health [49]. The authors suggested that it was possible that individuals with diminished wellness may be more likely to seek such services to address these areas of need. As we did not examine the bidirectionality of this relationship, we can only speculate on directionality using cross-sectional data, and suggest that future prospective data collection approaches be used to clarify the nature of these relationships.

In the present study, the dependent variable of frequency of land-based activities was derived from two survey questions querying activities related to hunting, fishing, or trapping, and gathering plants. In reality, the definition of land-based is much more expansive, and can include activities such as spiritual engagement or arts-based activities in land-based ceremonies. It is likely that specific facets of land-based activity frequency—such as food security, engaging in spiritual practices, or sharing teachings between community members—would differ. Although previous research with the APS indicates that the majority of respondents engage in such practices for either personal use or pleasure [10], the reasons for engaging in these practices could differ among those living on-reserve, or could change when more cultural practices are embedded within the current definition of land-based activities.

### Study Limitations and Future Directions

This study’s findings are limited to those who were contacted and willing to participate in the nationally based APS. There are noticeable gaps in the survey sample that could affect the generalizability of these results, such as those individuals living with no fixed address, those registered as living on-reserve but currently residing off-reserve, and those who could not be contacted by the survey administrators. For First Nations individuals living on-reserve, predictors of frequency of engagement in land-based activities could vary; thus, results of the present study should not be extended to these populations.

Future research may explore other predictors of land-based activities among various age groups and community sizes. The present study focused specifically on determining whether residing in a small community predicted the frequency of land-based activities; however, across population sizes—such as residing in a more urban location—predictors could differ. Although being male predicted the frequency of engagement in land-based activities, it is likely that more specific predictors of frequency differ between genders. For example, Kumar et al. [10] found that for women, being the sole parent in a multiple-child household negatively predicted any type of engagement in land-based activity; however, the frequency or duration of such activity was not considered in these analyses. It is possible that given traditional gender roles of who typically engages in specific ceremonies and traditional activities could potentially explain sex-based differences in the present study with respect to engagement in land-based activities. Combining activities such as hunting, fishing, and trapping with activities such as plant gathering may confound predictors of engagement frequency given different participation rates across males and females.

Furthermore, analyses can explore barriers to engaging in such activities as frequently as one desires. Although the current iteration of the APS (2017) assessed such frequency, it did not explore whether participants were engaging in these activities as much as they would like. It is possible that regular or more frequent engagement in these activities may serve as a protective factor to bolster mental health outcomes, particularly when such activities are associated with a strong sense of cultural belonging. As this study relied solely on self-reported data of participant estimations of frequency of engagement across a large timeframe, it is also possible that these reports of frequency may be inaccurate. Future studies could use different approaches that emphasize estimations of engagement in shorter durations (i.e., weekly). Similarly, as research indicates that single-item self-report estimates of physical and mental health are only moderately correlated with more well-established measures of these constructs, with noted differences between various ethnicities [62], refined measurement of these constructs—including the use of validated measures with Indigenous populations—may reduce such demand characteristics.

It is likely that there are different barriers to engagement in these activities based on sex, age, location, previous land-based experience, and cultural knowledge. A systematic review of land-based outcomes and the populations using them for either mental health intervention or wellness promotion could clarify how relationships differ between various populations. Currently, it appears that the majority of interventions described in the literature have either focused on culture camps [19,21,22], been youth-focused [21,23,24,26], or have explored culture as treatment within residential interventions for substance use [18,20], but to date, a comprehensive review of outcomes has not been completed. By understanding the established literature dedicated to this topic, and through more region- or community-specific studies of outcomes, the results of the present study can be more culturally contextualized and, potentially, more representative.

## 5. Conclusions

In reflecting on the importance of reconciliation, a renewed emphasis on Indigenous knowledge and ways of healing is needed. Colonialism in Canada has not only impacted Indigenous peoples’ engagement in land-based activities through the residential school system and broader attempts at cultural assimilation, but has simultaneously eroded Indigenous peoples’ relationships with the land through the reserve system and many instances of geographical displacement. This paper is a first attempt to better establish predictors of land-based activity engagement as a means to understand and promote mechanisms of cultural connectedness, meaning, and overall wellbeing going forward.

## Figures and Tables

**Table 1:** Participant demographics.

	Demographic	Frequency	Percent
Sex	Male	221,200	45.05
	Female	269,810	54.95
Age		*M* = 40.4	*sd* = 17.07
Life Stage	Youth (age 15 to 19)	62,570	12.74
	Adult	389,810	87.26
Marital Status	Single	207,470	42.25
	Married	140,990	28.73
	Living common law	79,940	16.28
	Separated	16,220	3.3
	Divorced	31,210	6.36
	Widowed	15,130	3.08
Place of Residence	Rural (under 1000)	112,930	23.00
	Small population (1000 to 29,999)	107,750	21.95
	Medium population (30,000 to 99,999)	72,860	14.84
	Large urban population (100,000 or greater)	197,410	40.21
Household Type	Two-generation	254,680	51.87
	Three or more generations	29,130	5.93
	Skip-generation	10,810	2.2
	Other household type	195,790	39.88
Highest Attained Education	Grade 8 or lower	24,050	4.90
	Some secondary education	64,130	13.06
	Secondary school diploma	73,560	14.98
	Some post-secondary	85,540	17.42
	Post-secondary diploma	150,460	30.64
	Bachelor’s degree	34,170	6.96
	Degree above bachelor level	14,730	3.00
Employment Status	Employed	267,820	54.54
	Unemployed	43,460	8.85
	Not in labor force ^1^	177,710	36.19
Estimated 2016 Total Personal Income	Less than CAD 5000	71,210	14.5
	CAD 5000 to 9999	34,470	7.02
	CAD 10,000 to 19,999	52,630	10.72
	CAD 20,000 to 29,999	45,850	9.34
	CAD 30,000 to 39,999	33,330	6.79
	CAD 40,000 to 49,999	27,550	5.61
	CAD 50,000 to 69,999	48,430	9.86
	CAD 70,000 or over	35,200	7.17
Residential School Attendance	Individual attendance	31,570	8.52
	Parent attendance	120,640	26.78
	Grandparent attendance	163,100	44.86

^1^ Individuals classified as “not in labor force” were those who identified themselves as retired, caring for children or family members, going to school, living with a chronic illness or disability, or not being able to find suitable work (i.e., wanting to work but not having any work available).

**Table 2:** Predictors of the frequency of land-based activity engagement for First Nations individuals living off-reserve.

Predictor	Block 1			Block 2			Block 3		
	*B*	*SE B*	*z*	*B*	*SE B*	*z*	*B*	*SE B*	*z*
Age	−0.001	0.002	−0.36	−0.002	0.002	−0.81	−0.002	0.002	−0.81
Sex (Female)	−0.26	0.07	−3.83 ^b^	−0.25	0.07	−3.66 ^b^	−0.24	0.07	−3.67 ^b^
Rural Living	1.29	0.11	10.76 ^b^	0.90	0.08	10.67 ^b^	0.90	0.09	10.47 ^b^
Physical Health				−0.03	0.04	0.70	0.02	0.04	0.65
Mental Health				0.05	0.04	1.26	0.05	0.04	1.23
Cultural Belonging				0.11	0.03	3.81 ^b^	0.11	0.03	3.96 ^b^
Individual RSA							−0.07	0.15	−0.47
Family RSA							−0.03	0.04	−0.63
Constant	1.29	0.11	11.56 ^b^	0.85	0.16	5.48 ^b^	0.88	0.16	5.46 ^b^
Wald Chi^2^	137.0 ^b^			154.0 ^b^			154.0 ^b^		
*R* ^2^	0.066			0.075			0.0755		

^a^*p* < 0.01; ^b^ *p* < 0.001.

## Data Availability

Restrictions apply to the availability of these data. Data were obtained from the CRDCN and are available with permission. Procedures to access these data can be found at https://crdcn.ca/, accessed on 25 April 2022.
